# Supplementary material for: A Global Screen for Assembly State Changes of the Mitotic Proteome by SEC-SWATH-MS
Source: Cell Syst. 2020 Feb 26;10(2):133–155.e6. doi: 10.1016/j.cels.2020.01.001 (PMC7042714; doi:10.1016/j.cels.2020.01.001)

P52564 | MP2K6_HUMAN | MAP2K6 MEK6 MKK6 PRKMK6 SKK3
Monomer MW [kDa]: 37.492 Monomer expected elution fraction: 49

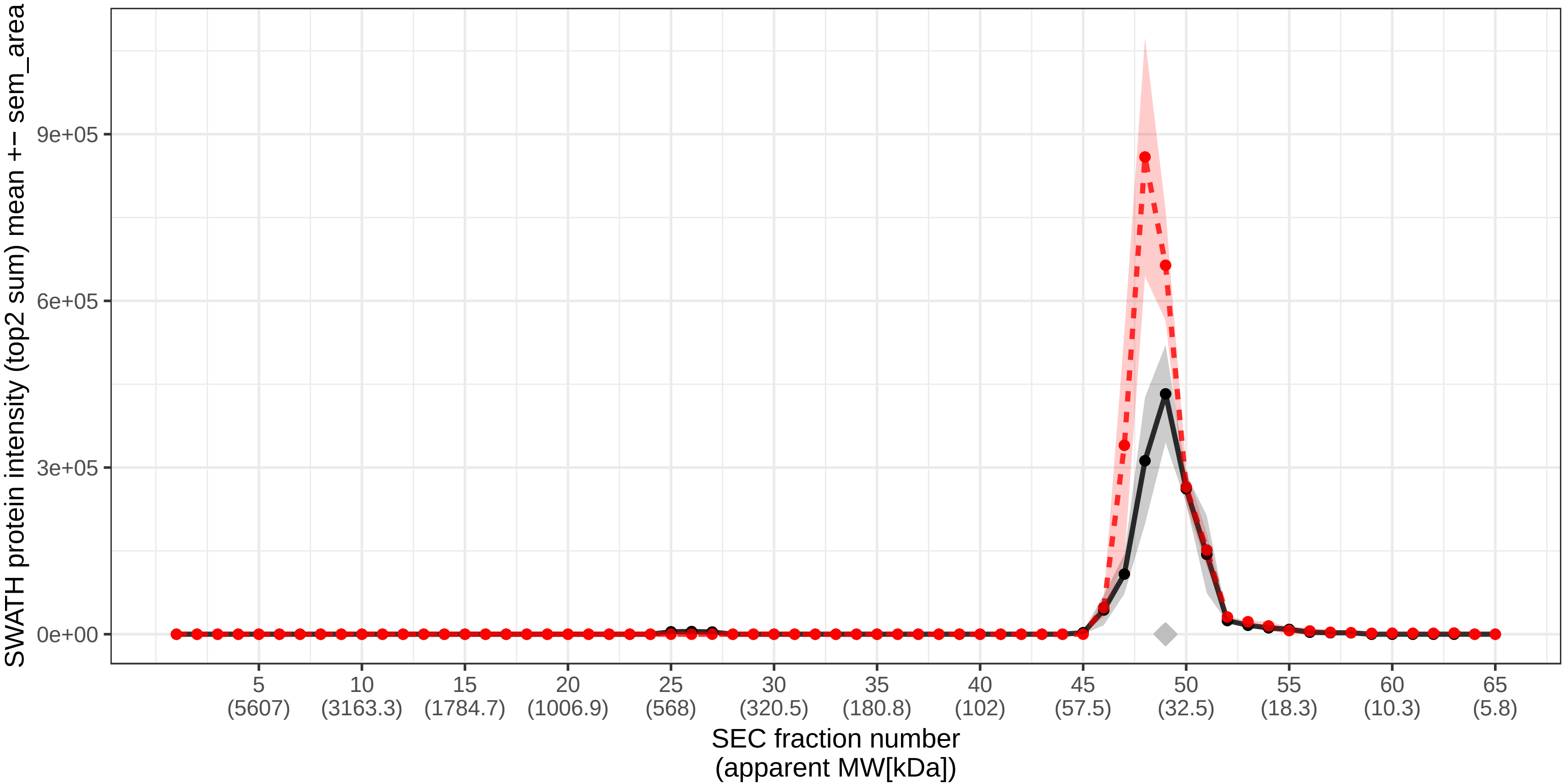

Supplement: Data S1. SEC-SWATH-MS Protein Chromatograms, Related to Figure 1 [file mmc6.zip › SECchrom_P52564_MP2K6_HUMAN_MAP2K6_MEK6_MKK6_PRKMK6_SKK3.pdf]
